# Alterations of gut microbiota in cirrhotic patients with spontaneous bacterial peritonitis: A distinctive diagnostic feature

**DOI:** 10.3389/fcimb.2022.999418

**Published:** 2022-09-06

**Authors:** Zumo Zhou, Hui Lv, Jiawen Lv, Yongming Shi, Heqing Huang, Lin Chen, Ding Shi

**Affiliations:** ^1^ Department of Infectious Diseases, Zhuji People’s Hospital of Zhejiang Province, Zhuji Affiliated Hospital of Wenzhou Medical University, Zhejiang, China; ^2^ Health Promotion Center, Zhejiang Provincial People’s Hospital, People’s Hospital of Hangzhou Medical College, Hangzhou, China; ^3^ State Key Laboratory for Diagnosis and Treatment of Infectious Diseases, National Clinical Research Center for Infectious Diseases, Collaborative Innovation Center for Diagnosis and Treatment of Infectious Diseases, Hangzhou, China; ^4^ Department of Infectious Diseases, The First Affiliated Hospital, College of Medicine, Zhejiang University School of Medicine, Hangzhou, China; ^5^ Shandong Laboratory, Jinan Microecological Biomedicine, Jinan, China

**Keywords:** spontaneous bacterial peritonitis, gut microbiota, 16S rRNA, diagnostic model, microbial marker

## Abstract

**Background:**

Spontaneous bacterial peritonitis (SBP) is a severe infection in cirrhotic patients that requires early diagnosis to improve the long-term outcome. Alterations in the gut microbiota have been shown to correlate with the development and progression of liver cirrhosis. However, the relationship between SBP and gut microbiota remains unknown.

**Methods:**

In this study, we applied 16S rRNA pyrosequencing of feces to ascertain possible links between the gut microbiota and SBP. We recruited 30 SBP patients, 30 decompensated cirrhotic patients without SBP (NSBP) and 30 healthy controls. Metagenomic functional prediction of bacterial taxa was achieved using PICRUSt.

**Results:**

The composition of the gut microbiota in the SBP patients differed remarkably from that in the NSBP patients and healthy individuals. The microbial richness was significantly decreased, while the diversity was increased in the SBP patients. Thirty-four bacterial taxa containing 15 species, mainly pathogens such as *Klebsiella pneumoniae*, *Serratia marcescens* and *Prevotella oris*, were dominant in the SBP group, while 42 bacterial taxa containing 16 species, especially beneficial species such as *Faecalibacterium prausnitzii*, *Methanobrevibacter smithii* and *Lactobacillus reuteri*, were enriched in the NSBP group. Notably, we found that 18 gene functions of gut microbiota were different between SBP patients and NSBP patients, which were associated with energy metabolism and functional substance metabolism. Five optimal microbial markers were determined using a random forest model, and the combination of *Lactobacillus reuteri*, *Rothia mucilaginosa*, *Serratia marcescens*, *Ruminococcus callidus* and *Neisseria mucosa* achieved an area under the curve (AUC) value of 0.8383 to distinguish SBP from decompensated cirrhosis.

**Conclusions:**

We described the obvious dysbiosis of gut microbiota in SBP patients and demonstrated the potential of microbial markers as noninvasive diagnostic tools for SBP at an early stage.

## Introduction

Spontaneous bacterial peritonitis (SBP) is a severe complication in patients with liver cirrhosis, and it is also an important cause of acute decompensated cirrhosis, with a 1-year mortality of up to 66% (Haderer et al., 2022). As the most common infection of end-stage liver diseases, the prevalence of liver cirrhosis has given rise to a rapidly increasing number of SBP cases, which may deteriorate into septic shock and multisystem organ failure apace, but patients could survive with early antimicrobial therapy. However, due to asymptomatic clinical symptoms in the early stage and a lack of noninvasive screening methods, over half of the patients have advanced-stage disease at their initial diagnosis, losing the opportunity for timely intervention (Marciano et al., 2019).Therefore, it is urgent to create a sensitive and accurate method for SBP screening in the early phase.

The gut and liver are closely linked anatomically and functionally, forming the so-called gut-liver axis, which means blood, rich in various foreign substances absorbed by intestine and metabolites of intestinal microorganisms, is recycled by the hepatic portal vein to be processed by the liver directly; reciprocally, the liver performs significant functions by enterohepatic circulation in the gut. Nevertheless, gut microbiota dysbiosis is one of the crucial causes of bacterial translocation (BT) resulting from pathologic crosstalk within the gut-liver axis, which in turn can interfere with major events of the pathophysiological cascade underlying decompensated cirrhosis (Marciano et al., 2019). The change in gut microbiota in liver cirrhosis patients has been widely studied. Our previous study revealed that *Veillonella*, *Streptococcus*, *Clostridium* and *Prevotella* were enriched in liver cirrhosis patients (Qin et al., 2014). Altered gut microbiota was associated with the progression of liver cirrhosis and became more severe in the decompensated stage (Bajaj et al., 2014). However, gut microbiota changes in SBP patients and the role of gut flora in the progression of SBP have not been fully revealed.

To date, a considerable number of studies about the application of gut microbiota as diagnostic biomarkers for liver diseases such as liver cirrhosis, autoimmune hepatitis and hepatocellular carcinoma (HCC) have been published (Qin et al., 2014; Ren et al., 2019; Rao et al., 2021) but have not addressed SBP. The present research evaluated the altered gut microbiota in SBP patients and the possible relationship between the gut microbiota and disease progression, and explored a specific gut microbiota profile as a biomarker panel and redound to establish a noninvasive diagnostic model in screening for SBP at an early stage.

## Methods

### Participant recruitment

All included patients were diagnosed with liver cirrhosis with ascites, with or without SBP, and were recruited from the Zhuji People's Hospital from January 1 to December 30, 2020. Each cohort in SBP or NSBP consisted of 30 patients, and 30 age- and gender-matched healthy controls were also recruited. The diagnosis of cirrhosis was based on clinical, biochemical, radiological (ultrasonography), and endoscopic findings (presence of varices) or liver histology. The diagnosis of SBP was based on a polymorphonuclear (PMN) cell count of 250 or more per cubic mm or culture positivity of ascitic fluid (European Association for the Study of the Liver, 2018). The inclusion criteria were the presence of decompensated liver cirrhosis and ascites fluid. The exclusion criteria were upper gastrointestinal bleeding, intake of antibiotic therapy in the previous 2 weeks, hepatocellular carcinoma, other associated causes of ascites (such as tubercular or malignant ascites), and severe cardiopulmonary or renal complications. This study was approved by the National Health Commission of China and the Ethics Commission of the Zhuji People's Hospital. Written informed consent was obtained from all participating patients.

### Laboratory confirmation

The demographics and clinicopathological data of participants were collected from hospital electronic medical records and direct interviews. All laboratory tests were performed according to the clinical care needs of the patients. Complete blood counts, coagulation profiles, inflammatory indices, including C-reactive protein (CRP) and procalcitonin (PCT), and serum biochemical parameters, including renal and liver function, creatine kinase, ammonia, inflammatory cytokines, lymphocyte count, and immunoglobulin, were tested for all patients at admission.

### Fecal sample collection, genomic DNA extraction, and 16S rRNA sequencing

All 90 fecal samples were collected from participants at admission, immediately frozen using liquid nitrogen, and stored at –80°C for subsequent work. First, total genomic DNA was extracted from the samples using the CTAB/SDS method. DNA concentration and purity were measured on 1% agarose gels. Based on the initial concentration, DNA was diluted to 1 ng/μL using sterile water. Second, 16S rRNA genes of distinct regions (16S V3-V4) were amplified using specific primers with barcodes. All PCRs were carried out with 15 μL of Phusion^®^ High-Fidelity PCR Master Mix (New England Biolabs), 0.2 μM forward and reverse primers, and approximately 10 ng of template DNA. Thermal cycling consisted of initial denaturation at 98°C for 1 min, followed by 30 cycles of denaturation at 98°C for 10 s, annealing at 50°C for 30 s, and elongation at 72°C for 30 s. Finally, the samples were incubated at 72°C for 5 min. The same volume of 1X loading buffer (containing SYBR green) was mixed with PCR products, and electrophoresis was performed on a 2% agarose gel for detection. PCR products were mixed in equidensity ratios. Then, the PCR products were purified with a Qiagen Gel Extraction Kit (Qiagen, Germany). Sequencing libraries were generated using the TruSeq^®^ DNA PCR-Free Sample Preparation Kit (Illumina, USA) following the manufacturer’s recommendations, and index codes were added. The library quality was assessed on the Qubit@ 2.0 Fluorometer (Thermo Scientific) and Agilent Bioanalyzer 2100 system. Finally, the library was sequenced on an Illumina NovaSeq platform, and 250 bp paired-end reads were generated. The names of the repository/repositories and accession number(s) can be found below: https://www.ncbi.nlm.nih.gov/, accession ID: PRJNA861246.

### Pyrosequencing data bioinformatics analysis

Paired-end reads were assigned to samples based on their unique barcode and then truncated by cutting off the barcodes and primer sequences. After assembly, chimeric sequences were removed using Usearch software based on the Uchime algorithm (Edgar et al., 2011). Sequence analysis was performed by UPARSE software (UPARSE v7.0.1001). Sequences with ≥97% similarity were assigned to the same OTUs. Representative sequences for each OTU were screened for further annotation. For each representative sequence, the Silva Database was used based on the Mothur algorithm to annotate taxonomic information. Furthermore, to study the phylogenetic relationships of different OTUs and the differences in the dominant species in different samples (groups), multiple sequence alignments were conducted using MUSCLE software (Version 3.8.31). OTU abundance information was normalized using a standard sequence number corresponding to the sample with the fewest sequences.

Alpha diversity and beta diversity were calculated with QIIME (Version 1.7.0) and visualized with R software (Version 2.15.3). The gene functions of the gut microbiota and the 16S rRNA gene sequences in Kyoto Encyclopedia of Genes and Genomes (KEGG) were predicted by PICRUSt. A random forest model by R 3.4.1 software was constructed for distinguishing between the SBP and NSBP groups, and the 10 most predominant genera were selected as candidate biomarkers based on importance values.

### Statistical analysis

Statistical analysis was performed using SPSS version 20.0 (SPSS Inc.). For most variables, descriptive statistics such as median with interquartile range (IQR; for data with skewed distribution) and proportion (%) were calculated. The Mann−Whitney U test was used to compare normally distributed variables; otherwise, one-way ANOVA followed by the Student-Newman−Keuls method was used. The Wilcoxon rank sum test combined with the Benjamini−Hochberg method was applied to compare bacterial taxa. Correlations between variables were computed using the Spearman rank correlation. The values are presented as the mean ± SEM if normally distributed; otherwise, the values are presented as the median (25th and 75th percentiles). P < 0.05 was considered significant.

## Results

### Patient characteristics

A total of 90 participants were recruited in this study, including 30 SBP patients and 30 NSBP patients with 30 gender- and age-matched healthy controls. The demographic and clinical characteristics of SBP or NSBP patients are summarized in **Table 1**. Both groups of patients were mainly elderly, with an average age of more than 60 years old. The median number of PMNs in SBP patients was 620, which was significantly higher than that in the NSBP group. Other baseline characteristics, such as liver function, inflammatory indices and lymphocyte count, were similar in both groups. Of note, most patients belonged to Child−Pugh class B (40%) and C (56.6%) with a high mean MELD score. Liver function indices, such as total bilirubin (TB), g-glutamyltransferase (GGT), aspartate aminotransferase (AST), alkaline phosphatase (ALP) and alanine aminotransferase (ALT), were significantly increased in SBP or NSBP patients compared with healthy controls (P < 0.01), as were the inflammatory indices CRP and PCT (P < 0.01).

**Table 1:** Clinical characteristics of patients.

Characteristic	SBP (N = 30)	NSBP (N = 30)	Controls (N = 30)	p_1_	p_2_	p_3_
Demographics, n (%)							
Age, median (IQR)	61 (52.8, 70)	60 (50, 65.5)	57 (55, 60.3)	0.11	0.43	0.37	
Male sex	23 (76.7)	23 (76.7)	20 (66.7)	0.39	0.39	1.0	
Initial laboratory findings, median (IQR)							
PMN, mm^3^	620 (382, 810)	100 (50, 152)	NA	NA	NA	0.000	
Leukocyte count, 10^9^/L	5.1 (3.7, 8.7)	4.7 (3.6, 6.7)	5.6 (5.1, 6.4)	0.24	0.17	0.93	
Neutrophil count, 10^9^/L	3 (1.9, 7.1)	3.7 (2.2, 4.8)	3.2 (2.8, 3.9)	0.87	0.48	0.77	
Albumin, g/L	25.5 (22.4, 29.9)	25.5 (22.4, 28.9)	46.5 (42.6, 47.8)	0.000	0.000	0.72	
ALT, U/L	34 (23.5, 78.5)	40 (21.3, 81)	19.5 (16.8, 30)	0.000	0.002	0.92	
AST, U/L	52.5 (34.8, 100.8)	68.5 (30.5, 114.5)	20 (16, 26)	0.000	0.000	0.61	
ALP, U/L	114 (88.3, 155)	111.5 (76.8, 139.3)	77 (67.8, 85)	0.000	0.000	0.70	
GGT, U/L	66.5 (30.8, 154.8)	89.5 (37.8, 232.8)	28 (17, 52.8)	0.004	0.000	0.35	
Total bilirubin, μmol/L	31.2 (11.9, 62.7)	36.1 (22.4, 90.9)	14.1(9.9, 16.7)	0.000	0.013	0.38	
Creatinine, μmol/L	84 (63.8, 103.8)	73.5 (62.3, 117.5)	68 (47.5, 72.3)	0.000	0.000	0.43	
Prothrombin time, s	16.4 (14, 20.7)	17.4 (14.7, 20.5)	10.8 (10.4, 11.1)	0.000	0.000	0.65	
CRP, mg/L	14.9 (5.9, 36.3)	15.9 (2.1, 31.4)	0.6 (0, 1.1)	0.000	0.000	0.32	
PCT, ng/mL	0.18 (0.13, 1.48)	0.26 (0.13, 1.23)	0.02 (0.01, 0.05)	0.000	0.000	0.86	
CD4+ T cells/μL	448 (230, 661)	383 (197, 724)	NA	NA	NA	0.76	
CD8+ T cells/μL	213 (100, 361)	164 (113, 250)	NA	NA	NA	0.47	
CD45+ T cells/μL	1410 (649, 1787)	883 (601, 1483)	NA	NA	NA	0.17	
Severity score							
CTP score	9 (8, 11.3)	10 (9, 11)	NA	NA	NA	0.43	
MELD score	15 (9.8, 19.3)	15 (9.8, 19)	NA	NA	NA	0.97	

Data are presented as the median (interquartile range) unless otherwise indicated. Values in boldface indicate statistical significance (P <.05). P value: p1, SBP vs. control; p2, NSBP vs. control; p3, SBP vs. NSBP. Abbreviations: SBP, cirrhosis patients with spontaneous bacterial peritonitis; NSBP, cirrhosis patients without spontaneous bacterial peritonitis; IQR, interquartile range; PMN, polymorphonuclear leukocyte; ALT, alanine transaminase; AST, aspartate aminotransferase; ALP, alkaline phosphatase; GGT, gamma-glutamyltransferase; CRP, C-reactive protein; PCT, procalcitonin; CTP, Child-Turcotte-Pugh; MELD, Model for End-Stage Liver Disease; NA, not applicable.

### Gut microbial diversity and richness analysis in SBP patients

**Figure 1:**
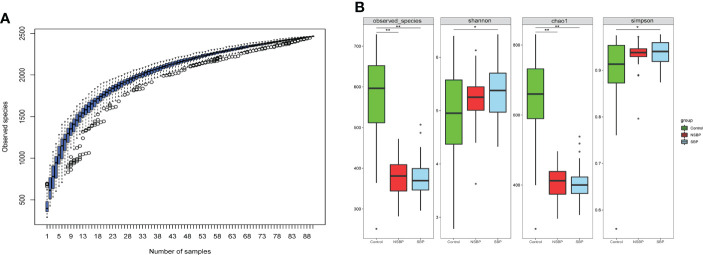
Comparison of the microbial richness and diversity in the SBP, NSBP and control groups (N = 30). **(A)** Specaccum species accumulation curves indicated a sufficient sampling size. **(B)** Compared with the control, microbial richness, which was calculated by observed species and Chao1, was significantly decreased in both SBP and NSBP patients (P < 0.001). Microbial diversity, which was characterized by the Shannon index and Simpson index, was significantly increased in SBP patients. No significant difference was observed between the SBP and NSBP groups (P > 0.05). The details of the indices above are listed in Supplementary Data S1. ^*^P < 0.05 and ^**^P < 0.01 vs. control in the SBP or NSBP group.

The specaccum species accumulation curve tended to be flat, indicating that nearly all species in the community were observed, which proved that the sample size was sufficient (**Figure 1A**). Compared with that of the controls, gut microbial richness, which was calculated by the observed species and the Chao1 index, was significantly decreased in SBP and NSBP patients (P < 0.001, **Figure 1B**). No significant difference was observed between SBP and NSBP patients in terms of microbial richness. In addition, both the Shannon index and Simpson index illustrated that the SBP patients were characterized by higher microbial community diversity than the healthy controls (P < 0.05). However, the overall microbial diversity was not significantly different between the NSBP patients and the controls. The details of the indices above are listed in **Supplementary Table 1**. This finding suggests that decompensated cirrhosis patients, with or without SBP, have obvious gut microbiota changes, which appear to be greater in patients with SBP.

### Compositional analysis of SBP and NSBP microbial clusters

**Figure 2:**
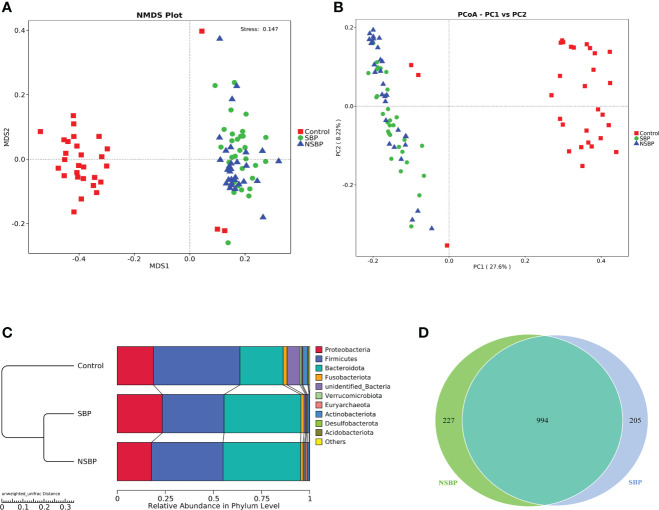
Comparisons of beta diversity in the SBP, NSBP and control groups (N = 30). A significant difference was found between the SBP group and the control group or the NSBP group and the control group by **(A)** NMDS analysis and **(B)** PCoA. No significant difference but a trend was found between the SBP and NSBP groups (ANOSIM; SBP vs. Control, R = 0.48, P = 0.001; NSBP vs. Control, R = 0.44, P = 0.001; SBP vs. NSBP, R = 0.03, P = 0.062). **(C)** Hierarchical clustering of different groups using Bray−Curtis dissimilarity indices at the phylum level by the unweighted UniFrac distances. **(D)** A total of 994 of the 1426 OTUs were shared between the SBP group and NSBP group by a Venn diagram. NMDS, nonmetric multidimensional scaling; PCoA, principal coordinate analysis.

To display the difference in bacterial communities between samples, beta diversity was calculated through nonmetric multidimensional scaling (NMDS) analysis and principal coordinate analysis (PCoA). The NMDS and PCoA analysis of unweighted UniFrac, which measured the phylogenetic similarities between microbial communities, showed a marked difference between the SBP or NSBP group and the control group (ANOSIM; SBP vs. control, R=0.48, P=0.001; NSBP vs. control, R=0.44, P=0.001; **Figure 2A**). In addition, the microbial structure of the SBP group showed a distinct trend from that of the NSBP group (ANOSIM; SBP vs. NSBP, R=0.03, P=0.062; **Figure 2B**). The average proportion of *Bacteroidota*, *Proteobacteria*, *Firmicutes*, *Fusobacteria* and *Actinobacteria* in the SBP and NSBP groups was up to 90% at the phylum level (**Figure 2C**). Furthermore, significant divergences of those five main phyla were observed between the SBP or NSBP groups and the control group. Most of the OTUs in the SBP and NSBP groups were similar, and 994 of the 1426 OTUs were shared between the SBP and NSBP groups by a Venn diagram (**Figure 2D**). Notably, 205 of 1426 OTUs were unique to SBP. Despite the highly diverse bacterial communities and interindividual differences, as shown in the heatmap of the relative abundances of the discrepant OTUs in each group (**Figure S1**), the intestinal microbial community of the cirrhotic patients were clearly affected by SBP.

### Comparisons of the gut microbiome in SBP and NSBP patients

**Figure 3:**
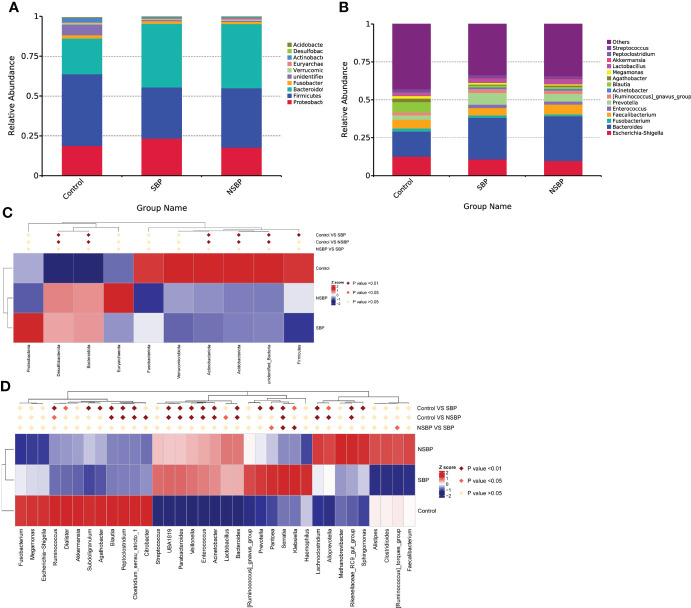
Composition and comparison of the gut microbiome in the SBP, NSBP and control groups. **(A)** The phylum-level and **(B)** genus-level composition diagrams show the composition characteristics of the different groups of gut microbiomes. Heatmap showing the abundance distribution of the OTUs identified as key variables among different groups. Metastat analysis showing the relative abundances of the significant bacteria at **(C)** the phylum level and **(D)** the genus level in patients compared with each group. We used the Wilcoxon rank-sum test to evaluate whether the difference in relative abundance was significant (dark red rhombus indicates P < 0.05; bright red rhombus indicates P < 0.01).

Statistical taxonomic analysis at the phylum and genus levels was carried out, and the average abundance of phyla and genera in groups was compared. As shown in the stacked bar plot at the phylum level, the bacterial phyla *Bacteroidota*, *Proteobacteria*, *Firmicutes*, *Fusobacteria* and *Actinobacteria* were the predominant taxa that made a difference in the three groups (**Figure 3A**). The group stacked bar plot at the genus level was also constructed (**Figure 3B**). Likewise, 15 genera, including *Escherichia-Shigella*, *Bacteroides*, *Fusobacterium*, *Faecalibacterium*, *Enterococcus*, *Prevotella* and *Akkermansia*, accounted for over 50% of the taxa among the samples in all groups. We adopted metastat analysis to measure the relative abundances of the significant bacteria at the phylum and genus levels among the 3 groups and presented the distribution of the OTUs identified as key variables by heatmaps. At the phylum level (**Figure 3C**), the relative abundances of the phyla *Bacteroidota* and *Desulfobacterota* significantly decreased in the control group compared with their relative abundances in the SBP and NSBP groups (P < 0.01). The relative abundances of the phyla *Firmicutes*, *Actinobacteriota* and *Acidobacteriota* were highest in the control group (P < 0.01). No significant difference was found between the SBP and NSBP groups at the phylum level. Meanwhile, at the genus level (**Figure 3D**), the healthy control group possessed the highest abundance of the genera *Ruminococcus*, *Blautia*, *Peptoclostridium* and *Clostridium_sensu_stricto* but the lowest quantity of the genera *UBA1819*, *Parabacteroides*, *Veillonella*, *Enterococcus*, *Acinetobacter*, *Bacteroides*, *Serratia*, *Lachnoclostridium* and *Rikenellaceae_RC9_gut_group* (P < 0.01). Notably, the SBP group had a relatively higher average abundance of the genera *Pantoea* (P < 0.05), *Serratia* (P < 0.01) and *Klebsiella* (P < 0.01) but a lower average abundance of the genus *Ruminococcus torques_group* (P < 0.05) than the NSBP group.

**Figure 4:**
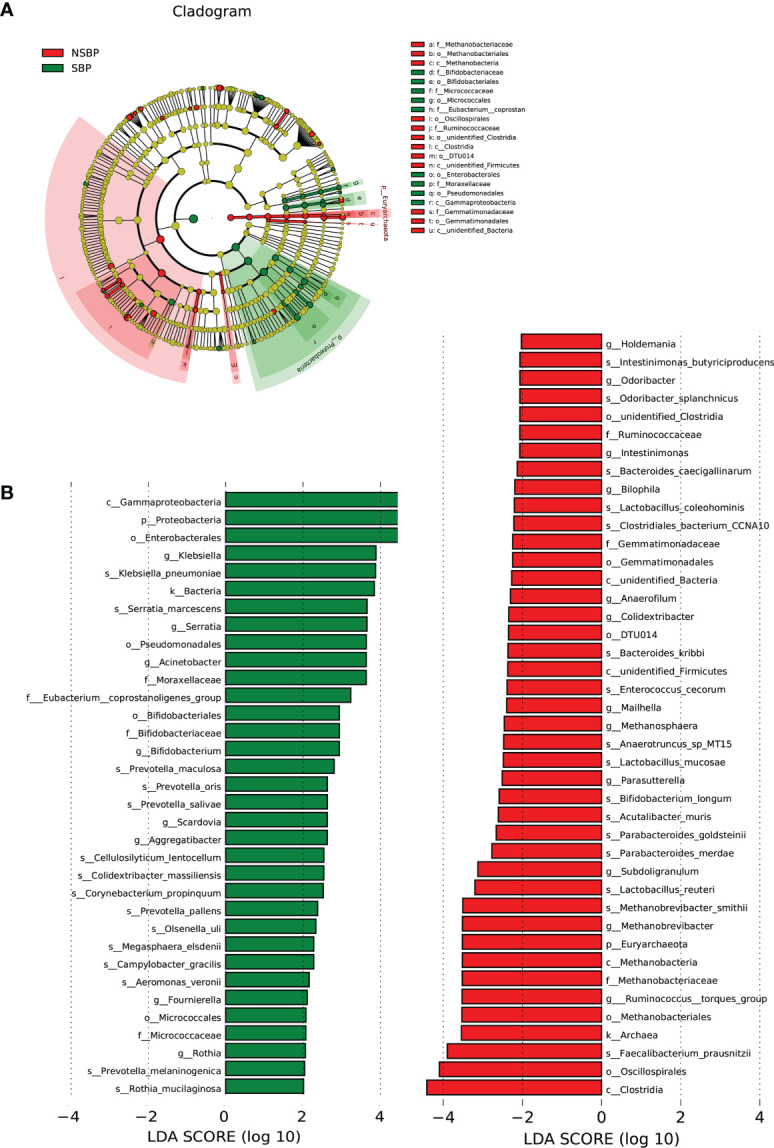
LEfSe and LDA analysis based on OTUs characterized the microbiome between the SBP and NSBP groups. **(A)** The cladogram shows the phylogenetic relationship between themicrobial taxa that were higher in the SBP patients than in the NSBP patients. **(B)** LDA scores showed significant bacterial differences between the SBP and NSBP groups. Only the taxa meeting a significant LDA threshold value of >2 are shown. Red indicates the NSBP group, and green indicates the SBP group. LDA, linear discriminant analysis; LEfSe, linear discriminant analysis effect size.

LEfSe was used to determine and distinguish the composition of the gut microbiome between the SBP and NSBP groups. The phylogenetic relationship between the two groups is shown in the phylogenetic tree with a cladogram to demonstrate the predominant taxa distribution associated with SBP (**Figure 4A**). Different taxa were further extracted and shown on a bar plot, which only presented significantly different species between the two groups with LDA scores greater than the preset value of 2 (**Figure 4B**). The length of the bar graph indicates the degree of influence of species that differ significantly between groups. The results revealed 34 taxa of the gut microbiome, including *Gammaproteobacteria*, *Proteobacteria*, *Enterobacterales*, *Klebsiella*, *Serratia*, *Acinetobacter*, and *Moraxellaceae*, which were extremely enriched (LDA>3) in the SBP group. For the NSBP group, 42 kinds of microbial biomarkers, including *Oscillospirales*, *Clostridia*, *Faecalibacterium prausnitzii*, *Ruminococcus torques group*, *Methanobrevibacter smithii* and *Lactobacillus reuteri*, were the predominant flora (LDA>3).

### Microbial gene function analysis in SBP patients

**Figure 5:**
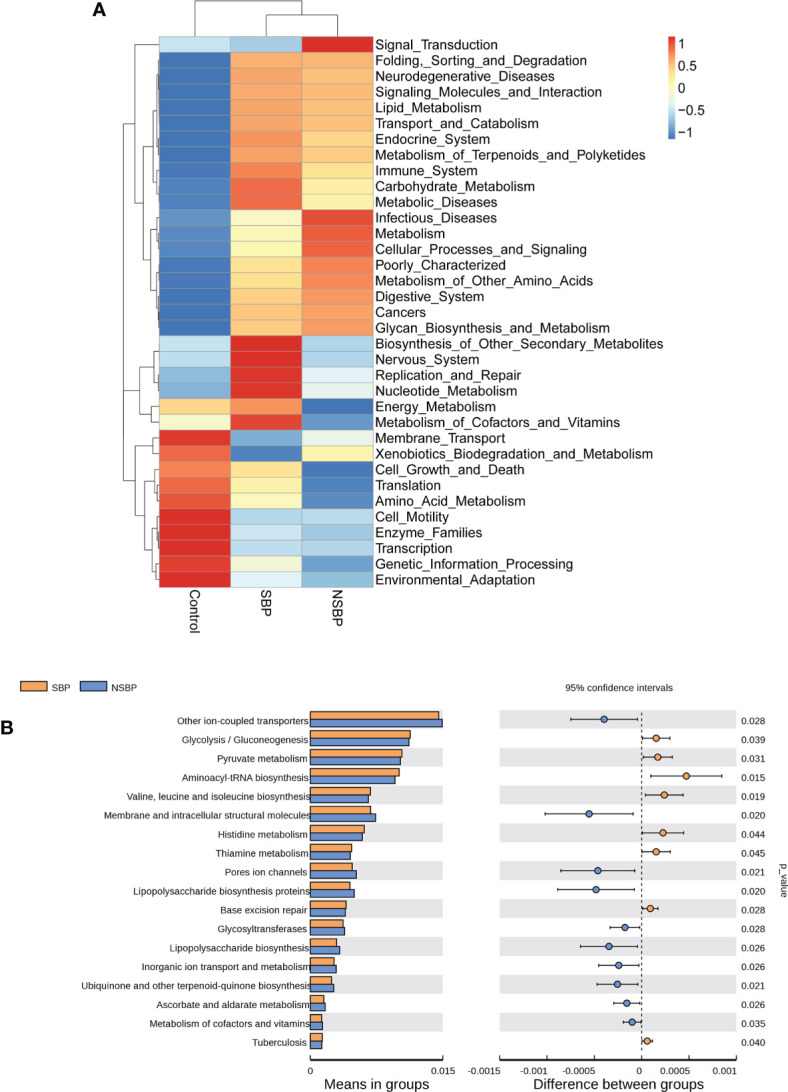
Heatmap visualizing the hierarchical clustering of the most significantly altered metabolites and metabolites related to the important pathways. **(A)** Top 35 most significant pathways identified from the KEGG pathway analysis of the significantly altered metabolites between the SBP group and NSBP group. The red (positive z score) and blue (negative z score) colors represent upregulation and downregulation, respectively. **(B)** Comparison of the most significant metabolic pathways between the SBP and NSBP groups. P < 0.05 is shown in the figure.

We used the PICRUSt tool to predict the abundances of functional categories of the KEGG ortholog (KO) based on closed-reference OTU picking to identify the metabolic and functional changes in the gut microbiota among all groups. A total of 35 pathways at level 2 with clearly differential abundances between SBP or NSBP patients and the controls are shown in the heatmap generated with the Z score (**Figure 5A**). There were 10 kinds of gene functions certified to be dominant in the control group, including membrane transport, xenobiotic biodegradation and metabolism, cell growth and death, translation, amino acid metabolism, cell motility, enzyme families, transcription, genetic information processing, and environmental adaptation. In addition, 18 pathways at level 3 were identified with significantly different abundances between SBP patients and NSBP patients(**Figure 5B**). Specifically, glycolysis/gluconeogenesis, pyruvate metabolism, aminoacyl-tRNA biosynthesis, valine, leucine and isoleucine biosynthesis, histidine metabolism, and thiamine metabolism showed higher activity in SBP-associated gut microbiota, while membrane and intracellular structural molecules, ion channel pores, lipopolysaccharide biosynthesis proteins, glycosyltransferases, ascorbate and aldarate metabolism and metabolism of cofactors and vitamins showed prominently decreased activity in the SBP-associated gut microbiota. Collectively, these data indicate that microbial functional dysbiosis may participate in the pathogenesis and development of SBP.

### Correlation analysis between differential bacterial species and clinical characteristics

**Figure 6:**
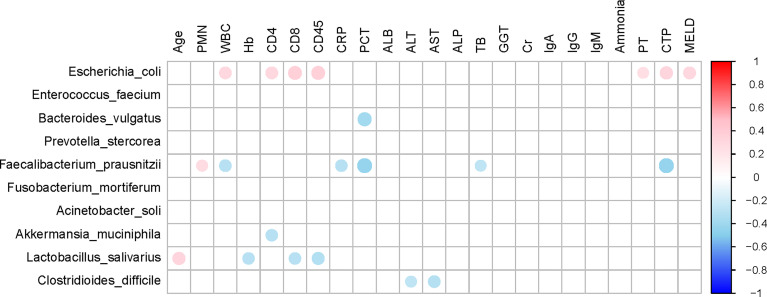
Associations between gut microbiomes and clinical indices of decompensated cirrhosis patients in SBP and NSBP groups. Distance correlation plots of the relative abundance of 10 species and clinical indices. Positive values (red) indicate positive correlations, and negative values (blue) indicate inverse correlations. Those correlations with a coefficient r < 0.25 are deleted and not shown in this figure. The color key and circle size indicate the strength of the correlation (r value).

The top 10 relative abundances of bacterial species were selected for correlation analysis with the clinical characteristics in SBP patients by the Spearman rank test (**Figure 6**). Notably, the abundance of *Escherichia coli* (9.8%) was positively correlated with WBC (P=0.02, r=0.30), CD4 count (P=0.03, r=0.31), CD8 count (P=0.01, r=0.36), CD45 count (P<0.01, r=0.37), PT (P=0.04, r=0.26), CTP (Child-Turcotte-Pugh) score (P=0.01, r=0.33) and MELD score (P=0.02, r=0.31). The abundance of *Faecalibacterium prausnitzii* (5.2%) decreased in SBP patients and was negatively correlated with WBC (P=0.02, r=-0.30), CRP (P=0.03, r=-0.29), PCT (P<0.01, r=-0.43), TB (P=0.04, r=-0.26), and CTP scores (P<0.01, r=-0.43). Moreover, the species *Clostridioides difficile* (1.1%) showed converse correlations with liver function, such as the levels of ALT (P=0.04, r=-0.26) and AST (P=0.02, r=-0.30). The abundance of *Bacteroides vulgatus* (11.8%) in the SBP patients was negatively correlated with the level of PCT (P<0.01, r=-0.38). The altered gut microbial profiles and their related host immunity and inflammation may actively participate in the pathophysiology of SBP.

### Diagnostic potential of SBP based on gut microbial markers

**Figure 7:**
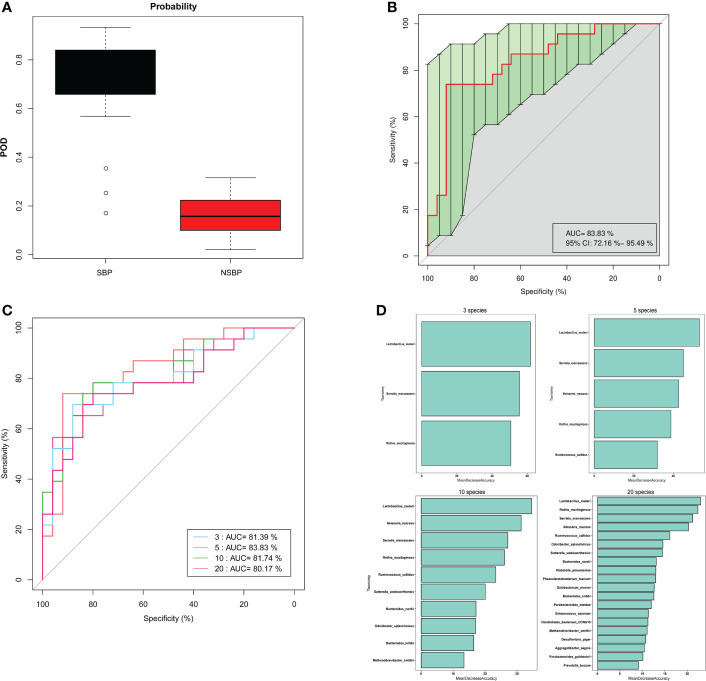
Gut microbiome as a noninvasive diagnostic model for SBP by the random forest model. **(A)** The POD value was significantly increased in SBP versus NSBP patients with a combination of five species. **(B)** The POD index achieved an AUC value of 83.8% with a 72.2% CI to 95.5% between SBP and NSBP patients. **(C)** ROC curve analysis in the SBP and NSBP cohorts combined to evaluate the classification ability of gut microbiome markers in predicting different groups. The combination of *Lactobacillus reuteri*, *Rothia mucilaginosa*, *Serratia marcescens*, *Ruminococcus callidus* and *Neisseria mucosa* reached a higher AUC value as the optimal biomarker. **(D)** Random forest classifier measured the importance of variable markers in the prediction of results. The mean decrease in accuracy for flora combinations of 3, 5, 10 and 20.

Based on the obvious change in the structure and composition of the gut microbiota between the SBP and NSBP groups, a random forest classifier model was constructed to assess the potential of gut microbial markers as a noninvasive diagnostic tool for SBP. The combination of five OTU-based biomarkers, *Lactobacillus reuteri*, *Rothia mucilaginosa*, *Serratia marcescens*, *Ruminococcus callidus* and *Neisseria mucosa*, were selected as the optimal set to distinguish the differences between 30 SBP patients and 30 NSBP patients. The probability of disease (POD) index was calculated using the data from the 5 selected microbial markers, and was significantly higher in SBP patients than in NSBP patients (**Figure 7A**). Of greater significance, in regard to the receiver operating characteristic (ROC) curve for the evaluation of the constructed models, the POD index reached an area under the curve (AUC) of 0.8383 with a 95% confidence interval (CI) of 0.7216–0.9549 (p < 0.0001, **Figure 7B**). When other diagnostic models of combinations with more or fewer bacterial species were constructed, the AUC value did not improve (**Figures 7C, D**). The data indicated that the classifier model based on the 5 specific microbial markers displayed powerful diagnostic potential in distinguishing SBP from decompensated cirrhosis patients.

## Discussion

SBP is a severe complication in patients with decompensated liver cirrhosis that requires rapid and accurate antibiotic treatment to improve clinical outcomes. Bacterial translocation (BT) from the gut to the mesenteric lymph nodes is recognized as a major mechanism for the pathogenesis of SBP (Haderer et al., 2022). Previous studies have confirmed that the gut microbiome is profoundly altered with relative overexpression of pathological species and loss of some keystone taxa in patients with cirrhosis (Qin et al., 2014). Moreover, gut microbiota dysbiosis is more pronounced in decompensated cirrhotic patients accompanying cirrhosis progression (Bajaj et al., 2014). The presence of bacterial DNA in ascites translocated from the intestine was directly associated with poor clinical outcomes in SBP patients (Fagan et al., 2015). This study demonstrated that an evident imbalance of the gut microbiome exists in SBP patients. Meanwhile, in contrast to NSBP patients with decompensated liver cirrhosis, the composition and structure of the gut microbiota was unique in SBP patients.

Based on the microbial composition of SBP patients, gut microbial richness in SBP and NSBP patients was significantly decreased compared to that in healthy controls. Consistent with our previous study, gut gene richness was much lower in patients with liver cirrhosis than in healthy individuals (Qin et al., 2014). This difference may be more pronounced because the cohort we enrolled this time consisted of only patients with decompensated cirrhosis. The loss of gut microbial richness was characterized by more overall adiposity and a significant inflammatory phenotype associated with obesity and ulcerative colitis (Le Chatelier et al., 2013; Wan et al., 2022). Surprisingly, the gut microbial diversity was higher in SBP patients than in healthy individuals. In previous studies of cirrhosis-related microbiota, no significant difference in bacterial diversity was found (Chen et al., 2011; Qin et al., 2014; Lv et al., 2016). Although gut microbial diversity tends to decrease in the majority of diseases, it is increased from cirrhosis to early HCC (Ren et al., 2019). We proposed that at the early stage of liver disease or infection, the microbial diversity may increase at first accompanied by enrichment of the more pathogenic bacteria.

The dysbiosis of gut microbiota in different digestive system diseases, such as CRC, HCC, and Inflammatory bowel disease (IBD), was characterized by their unique alteration of microbial composition. In this study, it was proven through NMDS and PCoA that there was a separated trend in communities of gut microbiota between SBP and NSBP patients. The difference in microbial composition of SBP patients was more obvious when compared to the HCs than the NSBP patients, indicating that the SBP microbiome has a more serious imbalance. Based on the LefSe and LDA score, 34 bacterial taxa, including 15 species, were dominant in the SBP group, while 42 microbial biomarkers containing 16 species were enriched in the NSBP group. Among them, some pathogenic bacteria, such as *Klebsiella pneumoniae*, *Serratia marcescens*, and *Prevotella oris*, were extremely enriched in SBP patients. Gram-negative bacteria were the main etiological agent of SBP, and *K. pneumoniae* was the most common cause, second only to *Escherichia coli* (European Association for the Study of the, L, 2010). *K. pneumoniae* may translocate from the intestine to cause liver infection (Lin et al., 2013). Clinical outcomes of SBP due to ESBL-producing *K. pneumoniae* are confirmed to be worse, with 30-day mortality rates ranging between 40 and 67% (Cheong et al., 2009; Kim et al., 2014). Consistent with another study, the gut microbiota of most septic patients was dominated by *Klebsiella*, indicating that the pathogens causing secondary infection in septic patients might originate from the intestinal colonization of pathogens (Mu et al., 2022). *S. marcescens* is also a common clinical infectious microorganism that causes septicaemia, pneumonia, urinary tract infection, endocarditis and arthritis acquired both in the community and the hospital (Su et al., 2003). In SBP patients, *S. marcescens* was more likely to lead to the invasion of the peritoneal cavity due to the weakened local resistance of the intestinal mucosa and imbalance of gut microbiota (Jimi et al., 1981). *P. oris* is an anaerobic bacterium mainly involved in oral cavity infections and leads to systemic infectious lesions such as empyema, cervical spinal epidural abscesses, and meningitis (Sato and Nakazawa, 2014). A case of septic shock caused by *P. oris* of hepatic origin was reported, which indicated its high virulence (Cobo et al., 2022). Correspondingly, some beneficial bacterial taxa were decreased in SBP patients, such as *Faecalibacterium prausnitzii*, *Methanobrevibacter smithii* and *Lactobacillus reuteri*. The species *F. prausnitzii* has been demonstrated to have a lower abundance in patients with liver cirrhosis than in healthy people, and is associated with the regression of acute-on-chronic liver failure (ACLF) (Wang et al., 2021; Chen et al., 2021). *F. prausnitzii* is the main butyrate-producing bacterium, and its short-chain fatty acid (SCFA) metabolites have an important anti-inflammatory effect on the intestine and liver (Lee et al., 2020). *M. smithii* is the dominant methanogen in the gut and can conserve energy through methanogenesis (Catlett et al., 2022). A decrease in *M. smithii* in gut communities was associated with IBD (Ghavami et al., 2018). *L. reuteri* serves as a probiotic to modulate periodontal parameters and subgingival biofilm dysbiosis by resolving inflammation and by reducing the molecular mediators associated with bone loss (Salinas-Azuceno et al., 2022). Furthermore, *L. reuteri* treatment was shown to protect liver function and recover liver superoxide dismutase concentrations in mice fed a Paigen atherogenic diet (Wang et al., 2022). Therefore, our study suggested a reduction in beneficial bacterial taxa accompanied by a significant increase in potential pathogens in SBP patients leads to a more obvious dysbiosis of gut microbiota, resulting in disruption of intestinal barrier function and bacterial translocation.

In this study, gene functions such as glycolysis/gluconeogenesis, pyruvate metabolism and aminoacyl-tRNA biosynthesis enriched in SBP patients were associated with energy metabolism, while the synthesis of functional substances that maintain cell structure and beneficial substances such as cofactors and vitamin metabolism were insufficient in SBP patients. These alterations in gene functions convincingly showed the potential influence of gut microbiota on metabolism in SBP patients, which indicated their contribution to the development of the disease (Engelmann et al., 2021). Recent advances have demonstrated a prevailing metabolic alteration in decompensated cirrhosis, especially in energy metabolism characterized by reduced oxidative glucose metabolism in the mitochondria and increased extramitochondrial glucose utilization through glycolysis (Moreau et al., 2020).

In addition, we analyzed the correlation between clinical indicators reflecting the severity of SBP disease and gut microbiota and found that the abundance of *F. prausnitzii* was negatively correlated with WBC, CRP, PCT, TB and CTP scores. The abundance of *E. coli* was positively correlated with WBC, CD4 count, CD8 count, CD45 count, PT, CTP score and MELD score. This finding provides theoretical support for the current clinical supplementation of probiotics or preventive use of antibiotics in SBP patients (Fernandez et al., 2016; Marciano et al., 2019). Although leukocyte esterase reagent strips (LERS) appeared to have a notable overall performance alternative to the cell count for the detection of SBP, there is currently a lack of non-invasive diagnostic method for SBP (Patel et al., 2022). Notably, five optimal microbial markers for SBP were identified by the random forest model. The microbial marker-based classifiers displayed a strong diagnostic potential in distinguishing SBP from decompensated cirrhotic patients with ascites. Early diagnosis and treatment for SBP in cirrhotic patients is critical to reduce mortality. Therefore, this study has important clinical application value in noninvasive diagnosis realized by the microbial mode for SBP.

Our study has some limitations. First, the study had a single-center retrospective design and a relatively small sample size, which may have compromised its statistical power. Second, we did not have a validation group. As gut microbial diagnosis can be influenced by many factors, such as regional, dietary, and population-based factors, it will be improved with cross-regional validation. Third, this study illuminated the characteristics of SBP and NSBP patients, but it cannot prove the causality between the imbalanced microbiota and the prognosis of the disease. More animal experiments and clinical trials are needed in the future.

## Conclusion

Our study suggests that the composition of the gut microbiota differs between SBP patients and NSBP patients. The dysbiosis of gut microbiota in SBP patients was more severe, with increased intestinal pathogenic bacteria and decreased beneficial bacterial taxa associated with disease severity. Five distinguished bacterial taxa have potential as biomarkers for SBP diagnosis in patients with decompensated cirrhosis. The random forest model achieved an accuracy of 83.83% with the combination of *Lactobacillus reuteri*, *Rothia mucilaginosa*, *Serratia marcescens*, *Ruminococcus callidus* and *Neisseria mucosa*.

## Data availability statement

The datasets presented in this study can be found in online repositories. The names of the repository/repositories and accession number(s) can be found below: NCBI, ID: PRJNA861246

## Ethics statement

The studies involving human participants were reviewed and approved by The Ethics Commission of the Zhuji People Hospital. The patients/participants provided their written informed consent to participate in this study.

## Author contributions

ZZ and DS designed the study. SY, HH, and LC collected clinical samples and performed the experiments. HL analyzed the data. DS wrote the manuscript. All authors contributed to the article and approved the submitted version.

## Funding

This work was supported by National Natural Science Foundation of China (82100643), the Zhejiang Provincial Natural Science Foundation of China (LGF19H030009, LQ20H030010), Research Project of Jinan Microecological Biomedicine Shandong Laboratory (JNL-2022001A).

## Conflict of interest

The authors declare that the research was conducted in the absence of any commercial or financial relationships that could be construed as a potential conflict of interest.

## Publisher’s note

All claims expressed in this article are solely those of the authors and do not necessarily represent those of their affiliated organizations, or those of the publisher, the editors and the reviewers. Any product that may be evaluated in this article, or claim that may be made by its manufacturer, is not guaranteed or endorsed by the publisher.

## Supplementary material

Supplementary Table 1 Comparison of diversity estimation of the 16S rRNA gene library at 97% similarity from the pyrosequencing analysis

Supplementary Figure 1 The heatmap shows the difference in the relative abundance of OTUs between SBP (N = 30) and NSBP (N = 30) at the phylum level. The relative abundance of each sample is shown. (Red indicates high abundance, and blue indicates low abundance).
