# Supplementary material for: Alterations of gut microbiota in cirrhotic patients with spontaneous bacterial peritonitis: A distinctive diagnostic feature
Source: Front Cell Infect Microbiol. 2022 Sep 6;12:999418. doi: 10.3389/fcimb.2022.999418 (PMC9485664; doi:10.3389/fcimb.2022.999418)
Supplement: Supplementary Table 1 — Comparison of diversity estimation of the 16S rRNA gene library at 97% similarity from the pyrosequencing analysis [file Table_1.docx]

Supplementary Table 1. Comparison of diversity estimation of the 16S rRNA gene library at 97% similarity from the pyrosequencing analysis

| Diversity index | Control | SBP | NSBP |
| --- | --- | --- | --- |
| Observed species | 576±108 | 375±48^**^ | 377±46^**^ |
| Shanon | 4.93±0.84 | 5.41±0.52^*^ | 5.23±0.48 |
| Chao1 | 647.8±123.5 | 407.2±51.3^**^ | 406.9±48.9^**^ |
| Simpson | 0.892±0.087 | 0.938±0.028^*^ | 0.933±0.322 |

All data are given as the mean ± SD. ^*^P < 0.05 and^**^P < 0.01 vs. Control in the SBP or NSBP group.
