# Supplementary figures and images for: Alterations of gut microbiota in cirrhotic patients with spontaneous bacterial peritonitis: A distinctive diagnostic feature
Source: Front Cell Infect Microbiol. 2022 Sep 6;12:999418. doi: 10.3389/fcimb.2022.999418 (PMC9485664; doi:10.3389/fcimb.2022.999418)

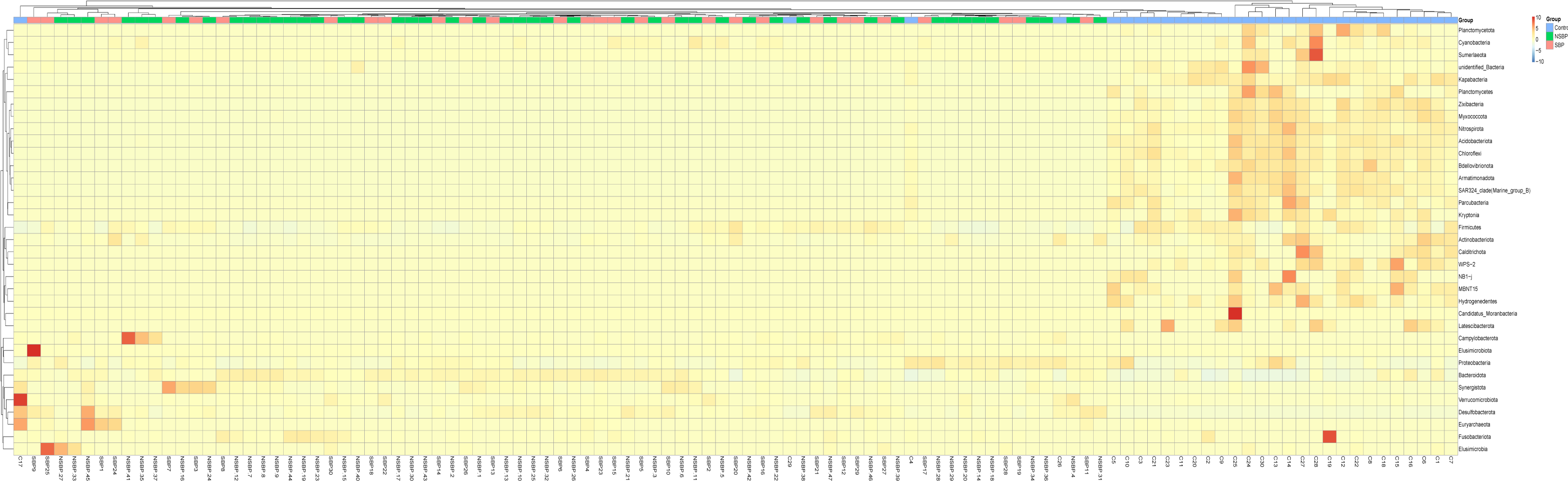

Supplement: Supplementary Figure 1 — The heatmap shows the difference in the relative abundance of OTUs between SBP (N = 30) and NSBP (N = 30) at the phylum level. The relative abundance of each sample is shown. (Red indicates high abundance, and blue indicates low abundance). [file Image_1.tif]
